# Supplementary material for: Updating and Refining of Economic Evaluation of Rotavirus Vaccination in Spain: A Cost–Utility and Budget Impact Analysis
Source: Viruses. 2024 Jul 25;16(8):1194. doi: 10.3390/v16081194 (PMC11360725; doi:10.3390/v16081194)
Supplement: Supplementary file 1 [file viruses-16-01194-s001.zip › Supplementary file S1/Table S1.Parameters used in the í░No vaccinationí▒ strategy.pdf]

Table S1. Parameters used in the “No vaccination” strategy.

PARAMETER	Definition	Base-case data	Data-collection time	Source	Country	Observations
**GENERAL POPULATION**						
**Rotavirus hospital admissions < 5 years**	Annual incidence per 1000 children < 5 years	3.2609	2000-2005	CMBD, *Conjunto Mínimo Básico de Datos* (Minimum Basic Data Set) [7]	Spain	An underdiagnosis of 50% is assumed. It is assumed that hospitalized cases came from primary or emergency care.
**Rotavirus nosocomial infection < 5 years**	Annual incidence per 1000 children < 5 years	0.8671	2000-2005	CMBD [7]	Spain	Calculated from proportion of nosocomial infections among rotavirus hospitalizations in CMBD, which was 26.59%
**Rotavirus infection with emergency care< 5 years**	Annual incidence per 1000 children < 5 years	18.90	2004-2005	Ardura-García 2021 [35], Van Damme 2007 [36]	Spain	
**Rotavirus infection with primary care < 5 years**	Annual incidence per 1000 children < 5 years	24.84	1997-2011	Ardura-García 2021 [35]	Germany, Italy	
**Rotavirus infection with healthcare < 5 years**	Annual incidence per 1000 children < 5 years	44.61	1997-2011	Calculated	Spain	Emergency + Primary care + Nosocomials
**Rotavirus infection without healthcare < 5 years**	Annual incidence per 1000 children < 5 years	178.43	1997-2011	Parashar 2003 [11]; Luquero 2008 [12]	Spain	Cases with healthcare multiplied by 4 in accordance with Parashar 2003 [11] and Luquero 2008 [12]
**Total rotavirus infections < 5 years**	Annual incidence per 1000 children < 5 years	223.04	1997-2011	Calculated	Spain	Healthcare + non-healthcare
**Probability of rotavirus healthcare**	Probability of receiving healthcare of a rotavirus infection in children < 5 years	0.20	1997-2011	Calculated	Spain	Ratio between healthcare and non-healthcare cases
**Probability of primary care**	Probability of primary care among those receiving healthcare in children < 5 years	0.5569	1997-2011	Calculated	Spain	Ratio between primary care and those receiving healthcare
**Probability of emergency care**	Probability of emergency care among those receiving healthcare in children < 5 years	0.4237	1997-2011	Calculated	Spain	Ratio between emergency care and those receiving healthcare
**Probability of nosocomial infection**	Probability of nosocomial infection among those receiving healthcare in children < 5 years	0.0194	1997-2011	Calculated	Spain	Ratio between nosocomial incidence and those receiving healthcare

**Hospital admission probability from emergencies**	Probability of being admitted to the hospital coming from emergencies in children < 5 years	0.10500	2016-2019	CMBD [7]	Spain	82.9% of hospitalizations come from emergencies (CMBD 2016-2019), the remainder from primary care
**Hospital admission probability from primary care**	Probability of being admitted to the hospital coming from primary care in children < 5 years	0.016479	2016-2019	CMBD [7]	Spain	82.9% of hospitalizations come from emergencies (CMBD 2016-2019), the remainder from primary care
**Lethality**	Rotavirus-attributable mortality per 100000 rotavirus hospital admissions	4.3653	2000-2019	CMBD [7]	Spain	Rotavirus-attributable deaths among those hospitalized because of rotavirus at all ages
**Rotavirus infection incidence 0-11 months**	Annual incidence of rotavirus infection per 1000 children aged 0 to 11 months	343.14	2000-2005	Díez-Domingo 2010 [19]	Spain	Calculated by applying the annual incidence in <5 years to the age distribution of Díez-Domingo 2010 [19]
**Rotavirus infection incidence 12-23 months**	Annual incidence of rotavirus infection per 1000 children aged 12 to 23 months	386.07	2000-2005	Díez-Domingo 2010 [19]	Spain	Calculated by applying the annual incidence in <5 years to the age distribution of Díez-Domingo 2010 [19]
**Rotavirus infection incidence 24-35 months**	Annual incidence of rotavirus infection per 1000 children aged 24 to 35 months	235.86	2000-2005	Díez-Domingo 2010 [19]	Spain	Calculated by applying the annual incidence in <5 years to the age distribution of Díez-Domingo 2010 [19]
**Rotavirus infection incidence 36-47 months**	Annual incidence of rotavirus infection per 1000 children aged 36 to 47 months	107.28	2000-2005	Díez-Domingo 2010 [19]	Spain	Calculated by applying the annual incidence in <5 years to the age distribution of Díez-Domingo 2010 [19]
**Rotavirus infection incidence 48-60 months**	Annual incidence of rotavirus infection per 1000 children aged 48 to 60 months	42.82	2000-2005	Díez-Domingo 2010 [19]	Spain	Calculated by applying the annual incidence in <5 years to the age distribution of Díez-Domingo 2010 [19]
**Rotavirus infection incidence 5-9 years**	Annual incidence of rotavirus infection per 1000 children aged 5 to 9 years	6.43	2000-2005	CMBD [7]	Spain	Calculated by applying the same probabilities of children < 5 years to the annual incidence found in CMBD [7]
**Rotavirus infectionincidence10-14years**	Annual incidence of rotavirus infection per 1000 children aged 10 to 14 years	1.45	2000-2005	CMBD [7]	Spain	Calculated by applying the same probabilities of children < 5 years to the annual incidence found in CMBD [7]

**Rotavirus infection incidence 15-44 years**	Annual incidence of rotavirus infection per 1000 aged 15 to 44 years	0.0544	2000-2005	CMBD [7]	Spain	Calculated by applying the same probabilities of children < 5 years to the annual incidence found in CMBD [7]
**Rotavirus infection incidence 45-64 years**	Annual incidence of rotavirus infection per 1000 aged 45 to 64 years	0.1222	2000-2005	CMBD [7]	Spain	Calculated by applying the same probabilities of children < 5 years to the annual incidence found in CMBD [7]
**Rotavirus infection incidence 65-74 years**	Annual incidence of rotavirus infection per 1000 aged 65 to 74 years	0.1516	2000-2005	CMBD [7]	Spain	Calculated by applying the same probabilities of children < 5 years to the annual incidence found in CMBD [7]
**Rotavirus infection incidence >74 years**	Annual incidence of rotavirus infection per 1000 aged >74 years	0.2842	2000-2005	CMBD [7]	Spain	Calculated by applying the same probabilities of children < 5 years to the annual incidence found in CMBD [7]
