# Supplementary material for: Updating and Refining of Economic Evaluation of Rotavirus Vaccination in Spain: A Cost–Utility and Budget Impact Analysis
Source: Viruses. 2024 Jul 25;16(8):1194. doi: 10.3390/v16081194 (PMC11360725; doi:10.3390/v16081194)
Supplement: Supplementary file 1 [file viruses-16-01194-s001.zip › Supplementary file S1/Table S2.Parameters used in the í░Targeted vaccinationí▒ strategy applied to the í░High-risk populationí▒ branch.pdf]

Table S2. Parameters used in the "Targeted vaccination" strategy applied to the "High-risk population" branch

PARAMETER	Definition	Base-case data	Data-collection time	Source	Country	Observations
**HIGH-RISK POPULATION**						
**Proportion of high-risk population**	Premature babies born between 25 and 32 week	0.009	2017-2019	Spanish Institute of Statistics (INE) [25]	Spain	
**Rotavirus hospital admissions < 5 years**	Annual incidence per 1000 children < 5 years	9.1304	2000-2001	Dennehy 2006 [16]	USA	Higher risk of rotavirus hospitalization among preterm babies (RR of 2.8)
**Rotavirus nosocomial infection < 5 years**	Annual incidence per 1000 children < 5 years	2.2804	2005	Herruzo 2009 [17]	Spain	Higher risk of nosocomial rotavirus infection among premature babies (OR 2.63)
**Rotavirus infection with emergency care< 5 years**	Annual incidence per 1000 children < 5 years	52.92	2000-2001	Dennehy 2006 [16]	USA	Higher risk of rotavirus hospitalization among preterm babies (RR of 2.8)
**Rotavirus infection with primary care < 5 years**	Annual incidence per 1000 children < 5 years	24.84	1997-2011	Ardura-García 2021 [35]	Germany, Italy	Same as general population
**Rotavirus infection with healthcare < 5 years**	Annual incidence per 1000 children < 5 years	80.04	1997-2011	Calculated	Spain	Emergency + Primary care + Nosocomials
**Rotavirus infection without healthcare < 5 years**	Annual incidence per 1000 children < 5 years	142.99	1997-2011	Calculated	Spain	Total minus healthcare cases
**Total rotavirus infections < 5 years**	Annual incidence per 1000 children < 5 years	223.04	1997-2011	Calculated	Spain	Same as general population
**Probability of rotavirus healthcare**	Probability of receiving healthcare of a rotavirus infection in children < 5 years	0.3589	1997-2011	Calculated	Spain	Ratio between healthcare and non-healthcare cases
**Probability of primary care**	Probability of primary care among those receiving healthcare in children < 5 years	0.3103	1997-2011	Calculated	Spain	Ratio between primary care and those receiving healthcare
**Probability of emergency care**	Probability of emergency care among those receiving healthcare in children < 5 years	0.6612	1997-2011	Calculated	Spain	Ratio between emergency care and those receiving healthcare
**Probability of nosocomial infection**	Probability of nosocomial infection among those receiving healthcare in children < 5 years	0.0285	1997-2011	Calculated	Spain	Ratio between nosocomial incidence and those receiving healthcare

**Hospital admission probability from emergencies**	Probability of being admitted to the hospital coming from emergencies in children < 5 years	0.107307	2016-2019	CMBD [7]	Spain	82.9% of hospitalizations come from emergencies (CMBD 2016-2019), the remainder from primary care
**Hospital admission probability from primary care**	Probability of being admitted to the hospital coming from primary care in children < 5 years	0.047156	2016-2019	CMBD [7]	Spain	82.9% of hospitalizations come from emergencies (CMBD 2016-2019), the remainder from primary care
