# Supplementary material for: Updating and Refining of Economic Evaluation of Rotavirus Vaccination in Spain: A Cost–Utility and Budget Impact Analysis
Source: Viruses. 2024 Jul 25;16(8):1194. doi: 10.3390/v16081194 (PMC11360725; doi:10.3390/v16081194)
Supplement: Supplementary file 1 [file viruses-16-01194-s001.zip › Supplementary file S2/Table S4.Results of the sensitivity analysis for probabilities of clinical events.pdf]

Table S4. Results of the sensitivity analysis for probabilities of clinical events. The comparison is universal vs. targeted vaccination from a societal perspective.

PARAMETER	Minimum value	Base-case data	Maximum value	ICUR Rotarix®. Base case €57,631/QALY	ICUR RotaTeq®. Base case€69,068/QALY	Sources and observations
**PROBABILITIES OF CLINICAL EVENTS. Unless specified, data used to estimate ranges come from references**						
**Primary care among those receiving healthcare**	0.29	0.5569	0.701	57631-57631	69067-69068	[7,24,36]
**Primary care among those receiving healthcare in the high-risk population**	0.18	0.3103	0.36	57631-57631	69067-69068	[3,16,17]
**Primary care among those receiving healthcare in the low-risk population**	0.2631	0.5608	0.7392	57631-57631	69067-69068	[24,16,17]
**Probability of being a home-care case**	0.666	0.8	0.888	50930-68560	62870-79089	The ranges of the Parashar model are expanded [11]. It is assumed that those who do not consult are double, 0.66; and the upper range is eight times more, 0.888.
**Home-care in the high-risk population**	0.32	0.6411	0.83	57376-57782	68838-69203	[24,16,17]
**Home-care in the low-risk population**	0.67	0.8014	0.88	32005-75875	42287-88135	[24,16,17]
**Hospitalization among those receiving primary care**	0.00990	0.01648	0.06486	57521-59560	68964-70878	[7,24]
**Hospitalization among those receiving primary care in the high-risk population**	0.03200	0.04716	0.13989	57602-57636	69041-69072	[7,24]
**Hospitalization among those receiving primary care in the low-risk population**	0.00710	0.01621	0.07777	50596-58689	61868-70150	[7,24]
**Hospitalization among those receiving emergency care**	0.08000	0.105	0.16143	56898-59294	68378-70632	[7,24]
**Hospitalization among those receiving emergency care in the high-risk population**	0.10010	0.107307	0.15629	57600-57636	69039-69072	[7,24]
**Hospitalization among those receiving emergency care in the low-risk population**	0.09000	0.10494	0.13992	54692-58897	66058-70364	[7,24]
**Nosocomial infection among those receiving healthcare**	0.011	0.0194	0.044319	57283-58670	68735-70058	[7]

**Nosocomial infection among those receiving healthcare in the high-risk population**	0.01200	0.0285	0.06376	57612-57640	69049-69076	[7]
**Nosocomial infection among those receiving healthcare in the low-risk population**	0.01010	0.0193	0.04341	54755-58744	66078-70224	[7]
**Emergency care among those receiving healthcare**	0.28	0.4237	0.69	55516-61571	67168-72605	[7,24]
**Emergency care among those receiving healthcare in the high-risk population**	0.62	0.6612	0.78	57610-57639	69049-69074	[7,24]
**Emergency care among those receiving healthcare in the low-risk population**	0.249	0.4199	0.703	46096-64712	57409-76224	[7,24]
**Proportion of high-risk population**	0.005	0.009	0.063	56590-57704	68135-69133	The maximum value is the percentage of preterm infants (<37 weeks) across the period 2017-2020. [15]
