# Supplementary material for: Updating and Refining of Economic Evaluation of Rotavirus Vaccination in Spain: A Cost–Utility and Budget Impact Analysis
Source: Viruses. 2024 Jul 25;16(8):1194. doi: 10.3390/v16081194 (PMC11360725; doi:10.3390/v16081194)
Supplement: Supplementary file 1 [file viruses-16-01194-s001.zip › Supplementary file S2/Table S5.Results of the sensitivity analysis for costs and other probabilities.pdf]

Table S5. Results of the sensitivity analysis for costs and other probabilities. The comparison is universal vs. targeted vaccination from a societal perspective.

PARAMETER	Minimum value	Base-case data	Maximum value	ICUR Rotarix®. Base case €57,631/QALY	ICUR RotaTeq® Base case €69,068/QALY	Sources and observations
**COSTS AND OTHER PROBABILITIES. Costs are in euros and updated in September 2021. If sufficient information is available, the ranges found in the literature are used. Otherwise, a reduction of 60% and an increase of 100% are used.**						
**Primary care costs SNHS perspective**	9.02	22.56	45.12	57048-57981	68439-69445	Minimum value is the Spanish value of Giaquinto [20]. Maximum value of Giaquinto is €90.06, which is an increase of 200%, so an increase of 100% is used.
**Hospitalization costs SNHS perspective**	2118.08	2315.06	2639.95	56804-58133	68206-69590	[7]
**Nosocomial infection costs SNHS perspective**	390.91	924.79	1446.37	57148-58125	68565-69582	[7,22]
**Nosocomial infections costs societal perspective**	132.59	270.83	404.59	57507-57759	68939-69201	[7,22]
**Emergency care costs SNHS perspective**	107.04	270.71	541.42	52391-60800	63426-72478	Minimum value is the Spanish value of Giaquinto [20]. Maximum value of Giaquinto is €636.89, which is an increase of 135%, so an increase of 100% is used.
**Primary care costs societal perspective**	79.09	197.73	395.46	52607-60646	63650-72318	Minimum value is a 60% reduction of the Spanish value of Giaquinto [20]. Maximum value of Giaquinto is €602.1, which is an increase of 155%, so an increase of 100% is used.
**Home care costs societal perspective**	31.89	53.15	106.3	47920-61516	59646-72836	General criteria. Reduction of 60% and increase of 100%.
**Hospitalization costs societal perspective**	341.19	402.08	804.16	56235-57843	67613-69288	Minimum value from Giaquinto [20]. Maximum value of Giaquinto is €964.7, which is an increase of 139%, so an increase of 100% is used.

**Emergency care costs societal perspective**	108.81	272.03	544.06	57631-62323	63997-72110	Minimum value is a 60% reduction of the Spanish value of Giaquinto [20]. Maximum value of Giaquinto is €670.34, which is an increase of 146%, so an increase of 100% is used.
**Rotarix®administration costs**	4.42	6	6.62	56885-57924		[21,35]
**RotaTeq® administration costs**	8.84	12	13.24		67577-69652	[21,35]
**Rotarix® price**	93.66	187.32	187.32	13408-57631		The minimum value is a 50% reduction in the price
**RotaTeq® price**	104.25	208.5	208.5		19900-69068	The minimum value is a 50% reduction in the price
**Efficacy/effectiveness of Rotarix® against rotavirus AGE**	0.46	0.74	0.87	43171-109257		Supplement 1
**Efficacy/effectiveness of RotaTeq® against rotavirus AGE**	0.5	0.735	0.87		53103-115689	Supplement 1
**Efficacy/effectiveness of Rotarix® against rotavirus AGE requiring healthcare**	0.22	0.03	0.03	56695-58035		Increase over AGE efficacy/effectiveness. Supplement 1
**Efficacy/effectiveness of RotaTeq® against rotavirus AGE requiring healthcare**	0.39	0.195	0.09		68631-69652	Increase over AGE efficacy/effectiveness. Supplement 1
**Efficacy/effectiveness of Rotarix® against rotavirus AGE requiring hospitalization**	0.06	0.08	0.07	57388-57956		Increase over AGE efficacy/effectiveness. Supplement 1
**Efficacy/effectiveness of RotaTeq® against rotavirus AGE requiring hospitalization**	0	0.015	0.03		68909-69120	Increase over AGE efficacy/effectiveness. Supplement 1
**Lethality among hospitalized**	0	0.000043653	0.0000873	57369-57896	68740-69398	[7]
**Increased hospitalization invagination**	0	0	0.0152000	57631-57765	69067-69183	[18]
**Vaccination coverage in the general population**	0.6	0.947	1	57604-57926	69040-69373	[13]
**Vaccination coverage increase in the high-risk population**	0	0.023	0.046	57627-57635	69064-69071	The maximum value is double that reported by Bruijning-Verhagen 2018 [14]
