# Supplementary material for: Updating and Refining of Economic Evaluation of Rotavirus Vaccination in Spain: A Cost–Utility and Budget Impact Analysis
Source: Viruses. 2024 Jul 25;16(8):1194. doi: 10.3390/v16081194 (PMC11360725; doi:10.3390/v16081194)
Supplement: Supplementary file 1 [file viruses-16-01194-s001.zip › Supplementary file S2/Table S6.Results of the sensitivity analysis for utilities and discounting.pdf]

Table S6. Results of the sensitivity analysis for utilities and discounting. The comparison is universal vs. targeted vaccination from a societal perspective.

PARAMETER	Minimum value	Base-case data	Maximum value	ICUR Rotarix®. Base case €57631/QALY	ICUR RotaTeq®. Base case €69068/QALY	Sources and observations
**DISCOUNTING AND UTILITIES. Unless specified, data used to estimate ranges come from references.**						
**Utility lost primary caregiver severe case**	-0.0064	-0.004	-0.0016	57155-58115	68473-69672	[22]
**Utility lost primary caregiver mild case**	-0.00203	-0.0014	-0.00087	51682-63811	62129-76229	Standard deviation provided by Hansen-Edwards [24], because no confidence interval is provided
**Utility lost primary caregiver moderate case**	-0.0023	-0.0014	-0.0003	55500-60.468	66320-72752	[22]
**Utility lost secondary caregiver severe case**	-0.0039	-0.0028	-0.0017	57412-57852	68794-69343	[22]
**Utility lost secondary caregiver mild case**	-0.00203	-0.0014	-0.00087	51682-63811	62129-76229	Standard deviation provided by Hansen-Edwards [24], because no confidence interval is provided
**Utility lost secondary caregiver moderate case**	-0.0027	-0.,0015	-0.0002	54825-61015	65452-73464	[22]
**Utility lost severe case**	-0.0053	-0.0034	-0.0015	57254-58014	68596-69546	[22]
**Utility lost mild case**	-0.0021	-0.0011	-0.000013	48728-71915	58668-85552	[23]
**Utility lost moderate case**	-0.0034	-0.0029	-0.0023	56428-59145	67513-71030	[22]
**Discounting rate**	0	3%	0.05	57270-57840	68677-69268	[37]
