# Supplementary material for: Updating and Refining of Economic Evaluation of Rotavirus Vaccination in Spain: A Cost–Utility and Budget Impact Analysis
Source: Viruses. 2024 Jul 25;16(8):1194. doi: 10.3390/v16081194 (PMC11360725; doi:10.3390/v16081194)
Supplement: Supplementary file 1 [file viruses-16-01194-s001.zip › Supplementary file S2/Table S7.Results of the sensitivity analysis for time horizon.pdf]

Table S7. Results of the sensitivity analysis for time horizon. The comparison is universal vs. targeted vaccination from a societal perspective.

TIME HORIZON	5 years	10 years	20 years	50 years	100 years (base case)
**ICUR Rotarix®. Base case €57631/QALY**	€58,001/QALY	€57,828/QALY	€57,751/QALY	€57,666/QALY	€57,631/QALY
**ICUR RotaTeq®. Base case €69068/QALY**	€69,517/QALY	€69,311/QALY	€69,217/QALY	€69,110/QALY	€69,068/QALY
