# Supplementary material for: Updating and Refining of Economic Evaluation of Rotavirus Vaccination in Spain: A Cost–Utility and Budget Impact Analysis
Source: Viruses. 2024 Jul 25;16(8):1194. doi: 10.3390/v16081194 (PMC11360725; doi:10.3390/v16081194)
Supplement: Supplementary file 1 [file viruses-16-01194-s001.zip › Supplementary file S3. systematic reviews.pdf]

Supplementary file 3.

Systematic Review

Methodology

A systematic review (SR) of the scientific literature was conducted in order to reply to the PICO research question shown in *Table 1*. The assessment of efficacy and safety was based on the results of randomized clinical trials; in every case, questions relating to effectiveness and safety were also supplemented with the results of observational studies. We also searched for economic studies as described below.

Table 1. Selection criteria

**Population**	Infants under 1 year in OECD countries(1)
**Intervention**	RotaTeq® or Rotarix® vaccination against rotavirus
**Comparators**	Placebo<br>No vaccination<br>At-risk population vaccination
**Outcomes**	• Number of episodes of rotavirus acute gastroenteritis (RV-AGE) and acute gastroenteritis (AGE) from any cause.<br>• Hospitalizations and severe cases of rotavirus infection.<br>• Health-related quality of life, utilities.<br>• Adverse events: cases of intussusception and other cases associated with vaccine administration.<br>• Rotavirus mortality<br>• Costs
**Study design**	For efficacy: randomized controlled clinical trials (RCTs), systematic reviews (SRs), meta-analyses and health technology assessment reports (HTARs).<br>For effectiveness: cohort studies, case-control studies, SRs, meta-analysis and HTARs.<br>For safety: RCTs, cohort studies, case-control studies, SRs, meta-analysis and HTARs.<br>For efficiency: economic evaluations, cost estimations.
**Language**	English and Spanish
**Publication date**	Until May 2021

The studies were selected according to the selection criteria shown in *Table 1*, but some studies were excluded if they met the following criteria:

● Studies duplicated or outdated by subsequent studies from the same institution.

● Narrative reviews, editorials, abstracts and communications to congresses, letters to the editor, and opinion articles.

The searches were performed in the following electronic databases:

- Medline (PubMed)
- Embase
- Web of Science (WOS)
- Scopus
- Cochrane Database of Systematic Reviews (Cochrane Library)
- Cochrane Central Database of Controlled Trials-Central
- Tripdatabase
- Prospero
- Database of Abstracts of Reviews of Effects (DARE), Health Technology Assessment (HTA) Database and National Health System Economic Evaluation Database (NHS-EED), Centre for Reviews and Dissemination (CRD)
- *Biblioteca Virtual en Salud*

Furthermore, the registry of clinical trials (clinicaltrials.gov) and gray literature were searched on the following websites:

- International Network of Agencies of Health Technology Assessment (INAHTA): http://www.inahta.org
- Canadian Agency for Drugs and Technologies in Health (CADTH): https://www.cadth.ca/
- Agency for Healthcare Research and Quality (AHRQ): http://www.ahrq.gov/research/index.html
- National Institute for Health and Care Excellence (NICE): https:// www.nice.org.uk
- United States Food and Drug Administration (FDA): www.fda.gov

For identification of studies, different search strategies were purpose-designed, adapted to each data source, by combining MESH terms and free text together with different Boolean and truncation operators. The strategies used in Medline, Embase, and Cochrane Library are described in **ANNEX 1**.

The study-selection process was carried out independently by peers. Any disagreements or differences of opinion were resolved by consensus or assistance of a third reviewer. Data extraction of the selected studies was performed by independent peer review.

A synthesis was performed by meta-analysis in cases where several studies were available for the same outcome. In the case of efficacy, all the variables for which meta-analysis could be performed were dichotomous. The summary relative risk with 95% confidence interval was calculated using a random-effects model. All analyses were performed using the RevMan 5.4 computer software program.

Results

The literature search identified 300 SRs on the efficacy, effectiveness, and safety of rotavirus vaccination. After elimination of duplicates, 232 references were selected for evaluation by title and abstract. Once the full-text review had been completed, 6 SRs were included, one SR on efficacy and safety, 2 SRs on effectiveness, and 3 SRs with observational studies on safety. The SR on efficacy and safety based on RCTs was a Cochrane review from 2019(2) with a high-quality rating,

according to our assessment using the AMSTAR 2 tool(3) (see **ANNEX 2** of this document). As its quality was judged to be sufficient, the abovementioned SR was updated with a literature search from April 2018 to the established time limit.

In terms of effectiveness analyses based on observational studies, the following 4 SRs met our selection criteria: Burnett, Wang, and Sun et al. (4-6). In the case of safety studies based on real-world data, we included the following 4 reviews which analyzed the safety of rotavirus vaccines: Gidengil, Rha, Kassim and Mellone(7-10). The quality assessment is available in **ANNEX 2**.

Summarized below are the efficacy, effectiveness, and safety data obtained by our review for the variables required in our economic assessment model (*Tables* 2 and *3*). In cases where several studies were available for the same variable and same study design, a meta-analysis was performed. The median of the results was selected for the base case in the Markov model and the ranges were used for the sensitivity analysis. Where results with a 1- and 2-year follow-up were available, the result with the longest follow-up was selected. When meta-analyses of case-control and cohort studies were available for the same variable, the results from cohort studies were selected in this summary because they provide higher-quality evidence.

Table 2. Summary of efficacy/effectiveness data considered for the model.

		Studies	N	RR/OR	95% CI
**Rotarix®**	RV-AGE any severity. 2-year follow-up	4 RCTs(11–14)	4,872	0.34	0.21-0.54
	RV-AGE any severity. Several follow-ups	9 case-control	46,857	0.18	0.13-0.25
	RV-AGE any severity. Summary	Median: 0.26. Range: (0.13-0.54)			
	RV-AGE with health care. 2-year follow-up	2 RCTs(14,15)	4,596	0.23	0.16-0.32
	RV-AGE with health care. Summary	Result: 0.23. Range: (0.16-0.32)			
	RV-AGE hospitalization. 2-year follow-up	4 RCTs(13–16)	19,243	0.17	0.11-0.26
	RV-AGE hospitalization. Several follow-ups	3 cohorts	553,743	0.16	0.12-0.22
	Severe RV-AGE. 2-year follow-up	5 RCTs(11,13–16)	19,442	0.13	0.09-0.21
	RV-AGE hospitalization and severe RV-AGE. Summary	Median: 0,15. Range: (0,09-0,26)			
**RotaTeq®**	RV-AGE any severity. 2-year follow-up	2 RCTs(17,18)	2,280	0.36	0.25-0.50
	RV-AGE any severity. Several follow-ups	8 case-control	14,539	0.17	0.13-0.21
	RV-AGE any severity. Summary	Median: 0.265. Range: (0.13-0.50)			
	RV-AGE with health care. 1-year follow-up	1 RCT(19)	57,134	0.07	0.04-0.11
	RV-AGE with health care. Summary	Result: 0.07. Range: (0.04-0.11)			
	RV-AGE hospitalization. 1-year follow-up	1 RCT(19)	57,134	0.04	0.02-0.10
	RV-AGE hospitalization. Several follow-ups	3 cohorts	194,113	0.07	0.04-0.11
	Severe RV-AGE. 2-year follow-up	2 RCTs(18,19)	2,596	0.06	0.01-0.39
	RV-AGE hospitalization and severe RV-AGE. Summary	Median: 0.055. Range: (0.01-0.39)			

95% CI: 95% confidence interval. RCT: Randomized controlled clinical trial. RV-AGE: rotavirus acute gastroenteritis

The systematic review about safety also identified RCTs and observational studies. Furthermore, a meta-analysis was performed in cases where there were several studies involving the same variable and/or with the same study design. *Table 3* shows a summary of the safety results.

Table 3. Safety results summary

	RCTs		Observational studies	
	Rotarix®	RotaTeq®	Cohorts	Case-control
**Mortality**	RR=1.25 (95% CI: 0.85–1.84) (9 RCTs) (11–16,20–22)	RR=1.24 (95% CI: 0.69–2.22) (6 RCTs) (17–19,23–25)	RR=1.05 (95% CI: 0.82-1.35) (14 studies) (7)	
**Intussusception**	RR=0.89 (95% CI: 0.34–2.30) (10 RCTs)(12–16,20–22,26,27)	RR=0.92 (95% CI: 0.84–1.01) (5 RCTs) (17,18,23,24,28)	RR=3.47 (95% CI: 1.23-9.78) (7studies)(8)	OR=1.,59 (95% CI: 1.11-2.27) (4 studies) (8)
			RA=1 to 6 cases per 100,000 infants (8 studies)(9)	
			RR=0.65 (95% CI: 0.41–1.05) (19 studies) (7)	
**Severe adverse events**	RR=0.86 (95%CI: 0.79-0.93) (12 RCTs)(11-16,21,22,27,28,30,31)	RR=0.69 (95% CI: 0.35–1.38) (9 RCTs) (17–19,22–24,27,30,31)	ND (asthma, autoimmune diseases, diabetes, seizures, encephalitis, thrombocytopenic purpura, stroke, kawasaki disease, meningitis, etc.) (43 studies) (7)	

95% CI: 95% confidence interval. ND: no difference between groups. OR: Odds ratio. RCTs: Randomized clinical trials. RR: Risk ratio.

With regard to economic studies, the literature search identified an SR published in 2017 by Kotirum et al. that answered our research question and included 104 economic assessments(3). This review with a search made up to November 2015, included 12 international studies and 92 studies conducted in a single country. The quality of Kotirum et al´s SR was deemed to be sufficient after evaluation with the AMSTAR 2 tool(3) (see quality assessment in Annex 2). We updated this SR, by identifying a total of 355 references after November 2015. Screening for duplicates and a peer review of the title and abstract resulted in the exclusion of 328 studies. The results of this screening, together with the studies included in the Kotirum´s SR, yielded a set of 117 economic assessments, which were then evaluated to select those of high relevance and applicability to our context. The ISPOR tool was used for the analysis of relevance and applicability(33). This analysis resulted in a selection of 16 studies of high relevance and applicability to our context and research questions: of these, 13 were included in the Kotirum SR and 3 were published thereafter. The main characteristics and results of these studies are summarized in *Table 4*. We extracted data on country, ICUR, threshold, author’s conclusions, and vaccination status in the countries concerned.

Table 4. Economic evaluations identified and selected. Unless specified, the evaluations compare universal vs. no vaccination.

Study	Country	ICUR (€/QALY) Base case	ICUR threshold (€/QALY)	Author's conclusions	Benefit status
**Bilcke 2009(34)**	Belgium	Rotarix®: 7,575<br>RotaTeq®: 30,227	50,000	Cost-effective	Universal vaccination
**Perez-Rubio 2011(35)**	Spain	Rotarix®: 23,435<br>RotaTeq®: 45,624	30,000	Cost-effective only Rotarix®	Vaccination of at-risk population
**Imaz 2014(36)**	Spain	RotaTeq®: 210,197	30,000	Not cost-effective	Vaccination of at-risk population
**Yamin 2016(37)**	France	RotaTeq®: 39,500	50,000	Cost-effective	Not included
**Goossens 2008(38)**	The Netherlands	Rotarix®: 28,488	Not defined	Cost-effective	Vaccination of at-risk population
**Rozenbaum 2011(39)**	The Netherlands	RotaTeq®: 46,717	Not defined	Inconclusive	Vaccination of at-risk population
**Bruijning-Verhagen 2018(40)**	The Netherlands	Both vaccines<br>51,277 (universal vs. no vaccination)<br>149,282 (universal vs. targeted vaccination)	Not defined	Inconclusive	Vaccination of at-risk population
**Tilson 2011(41)**	Ireland	Rotarix®: *Dominant	20,000	Cost-effective	Universal vaccination
**Chodick 2009(42)**	Israel	Rotarix®: 8,328<br>RotaTeq®: 23,233	23,540	Cost-effective	Universal vaccination
**Panatto 2009(43)**	Italy	Rotarix®: -41,114 (Dominant)	50,000	Cost-effective	Universal vaccination
**Sato 2011(44)**	Japan	6,800 (both vaccines)	47,236	Cost-effective	Universal vaccination
**Itzler 2013(45)**	Japan	RotaTeq®: 18,561	46,000	Cost-effective	Universal vaccination
**Hansen-Edwards 2017(46)**	Norway	Rotarix®: 47,447<br>RotaTeq®: 52,709	73,444	Cost-effective	Universal vaccination
**Milne 2009(47)**	New Zealand	RotaTeq®: 26,584	Not defined	Cost-effective	Universal vaccination
**Jit 2007(48)**	United Kingdom	Rotarix®: 86,522<br>RotaTeq®: 113,377	42,500	Not cost-effective	Universal vaccination
**Martin 2009(49)**	United Kingdom	Rotarix®: 16,676	29,100-42,500	Cost-effective	Universal vaccination

References

1. OECD. Our global reach - OECD. Member countries [Internet]. 2021 [cited 2021 Jan 21]. Available from: https://www.oecd.org/about/members-and-partners/

2. Soares-Weiser K, Bergman H, Henschke N, Pitan F, Cunliffe N. Vaccines for preventing rotavirus diarrhoea: vaccines in use. Cochrane Database Syst Rev [Internet]. 2019/11/07. 2019;2019(10). Available from: https://www.ncbi.nlm.nih.gov/pubmed/31684685

3. Kotirum S, Vutipongsatorn N, Kongpakwattana K, Hutubessy R, Chaiyakunapruk N. Global economic evaluations of rotavirus vaccines: A systematic review. Vaccine. 2017;35(26):3364‑86. Available from: https://www.ncbi.nlm.nih.gov/pubmed/28504193

4. Burnett E, Parashar U, Tate J. Rotavirus Vaccines: Effectiveness, Safety, and Future Directions. Paediatr Drugs [Internet]. 2018/02/02. 2018;20(3):223‑33. Available from: https://www.ncbi.nlm.nih.gov/pubmed/29388076

5. Wang H, Naghavi M, Allen C, Barber RM, Carter A, Casey DC, et al. Global, regional, and national life expectancy, all-cause mortality, and cause-specific mortality for 249 causes of death, 1980‑2015: a systematic analysis for the Global Burden of Disease Study 2015. Lancet [Internet]. 2016 Oct 8 [cited 2021 Jan 21];388(10053):1459‑544. Available from: /pmc/articles/PMC5388903/?report=abstract

6. Sun Z-WW, Fu Y, Lu H-LL, Yang R-XX, Goyal H, Jiang Y, et al. Association of Rotavirus Vaccines with Reduction in Rotavirus Gastroenteritis in Children Younger Than 5 Years: A Systematic Review and Meta-analysis of Randomized Clinical Trials and Observational Studies. JAMA Pediatr. 2021;175(7):e210347. Available from: https://www.embase.com/search/results?subaction=viewrecord&id=L635060300&from=export

7. Gidengil C, Goetz MB, Newberry S, Maglione M, Hall O, Larkin J, et al. Safety of vaccines used for routine immunization in the United States: An updated systematic review and meta-analysis. Vaccine. 2021;39(28):3696‑716. Available from: http://ovidsp.ovid.com/ovidweb.cgi?T=JS&PAGE=reference&D=medl&NEWS=N&AN=34049735

8. Kassim P, Eslick GD. Risk of intussusception following rotavirus vaccination: An evidence based meta-analysis of cohort and case-control studies. Vaccine. 2017 Jul 24;35(33):4276‑86.

9. Rha B, Tate JE, Weintraub E, Haber P, Yen C, Patel M, et al. Intussusception following rotavirus vaccination: An updated review of the available evidence. Expert Rev Vaccines. 2014;13(11):1339‑48. Available from: https://www.tandfonline.com/doi/abs/10.1586/14760584.2014.942223

10. Mellone NG, Silva MT, Paglia MDG, Lopes LC, Barberato-Filho S, Del Fiol F de S, et al. Kawasaki Disease and the Use of the Rotavirus Vaccine in Children: A Systematic Review and Meta-Analysis. Vol. 10, Frontiers in pharmacology. 2019. 1075 p.

11. Bernstein DI, Sack DA, Rothstein E, Reisinger K, Smith VE, O'Sullivan D, et al. Efficacy of live, attenuated, human rotavirus vaccine 89-12 in infants: a randomised placebo-controlled trial. Lancet. 1999/08/10. 1999;354(9175):287‑90. Available from: https://www.ncbi.nlm.nih.gov/pubmed/10440305

12. Salinas B, Schael IP, Linhares AC, Ruiz Palacios GM, Guerrero ML, Yarzabal JP, et al. Evaluation of Safety, Immunogenicity and Efficacy of an Attenuated Rotavirus Vaccine, RIX4414. Pediatr Infect Dis J. 2005;24(9):807‑16. Available from: https://pubmed.ncbi.nlm.nih.gov/16148848/

13. Vesikari T, Karvonen A, Puustinen L, Zeng SQ, Szakal ED, Delem A, et al. Efficacy of RIX4414 live attenuated human rotavirus vaccine in Finnish infants. Pediatr Infect Dis J

[Internet]. 2004;23(10):937‑43. Available from: https://pubmed.ncbi.nlm.nih.gov/15602194/

14. Vesikari T, Karvonen A, Prymula R, Schuster V, Tejedor JC, Cohen R, et al. Efficacy of human rotavirus vaccine against rotavirus gastroenteritis during the first 2 years of life in European infants: randomised, double-blind controlled study. Lancet. 2007/11/27. 2007 Nov 24;370(9601):1757‑63. Available from: https://www.ncbi.nlm.nih.gov/pubmed/18037080

15. Kawamura N, Tokoeda Y, Oshima M, Okahata H, Tsutsumi H, Van Doorn LJ, et al. Efficacy, safety and immunogenicity of RIX4414 in Japanese infants during the first two years of life. Vaccine. 2011/06/07. 2011 Aug 26;29(37):6335‑41. Available from: https://www.ncbi.nlm.nih.gov/pubmed/21640780

16. Ruiz-Palacios GM, Perez-Schael I, Velazquez FR, Abate H, Breuer T, Clemens SCAC, et al. Safety and efficacy of an attenuated vaccine against severe rotavirus gastroenteritis. N Engl J Med. 2006/01/06. 2006;354(1):11‑22. Available from: https://pubmed.ncbi.nlm.nih.gov/16394298/

17. Iwata S, Nakata S, Ukae S, Koizumi Y, Morita Y, Kuroki H, et al. Efficacy and safety of pentavalent rotavirus vaccine in Japan: a randomized, double-blind, placebo-controlled, multicenter trial. Hum Vaccin Immunother. 2013;9(8):1626‑33. Available from: https://www.ncbi.nlm.nih.gov/pubmed/23732903

18. Vesikari T, Matson DO, Dennehy P, Van Damme P, Santosham M, Rodriguez Z, et al. Safety and efficacy of a pentavalent human-bovine (WC3) reassortant rotavirus vaccine. N Engl J Med. 2006/01/06. 2006;354(1):23‑33. Available from: https://www.ncbi.nlm.nih.gov/pubmed/16394299

19. Vesikari T, Clark HF, Offit PA, Dallas MJ, DiStefano DJ, Goveia MG, et al. Effects of the potency and composition of the multivalent human-bovine (WC3) reassortant rotavirus vaccine on efficacy, safety and immunogenicity in healthy infants. Vaccine. 2006;24(22):4821‑9. Available from: https://www.ncbi.nlm.nih.gov/pubmed/16621194

20. Glaxo Smith Kline. A phase IIIb, double-blind, randomized, placebo-controlled, multicentre study to assess the immunogenicity, safety and reactogenicity of 2 doses of GlaxoSmithKline (GSK) Biologicals’ oral live attenuated human rotavirus (HRV) vaccine in healthy infants. 2 [Internet]. www.gsk-studyregister.com/study/2714: Glaxo Smith Kline. Available from: http://www.gsk-studyregister.com/study/2714

21. Omenaca F, Sarlangue J, Szenborn L, Nogueira M, Suryakiran P V, Smolenov I V, et al. Safety, reactogenicity and immunogenicity of the human rotavirus vaccine in preterm European Infants: a randomized phase IIIb study. Pediatr Infect Dis J. 2012;31(5):487‑93. Available from: https://www.ncbi.nlm.nih.gov/pubmed/22228231

22. Vesikari T, Karvonen A, Bouckenooghe A, Suryakiran P V., Smolenov I, Han HH. Immunogenicity, reactogenicity and safety of the human rotavirus vaccine RIX4414 oral suspension (liquid formulation) in Finnish infants. Vaccine. 2011;29(11):2079‑84. Available from: https://www.ncbi.nlm.nih.gov/pubmed/21238572

23. Block SL, Vesikari T, Goveia MG, Rivers SB, Adeyi BA, Dallas MJ, et al. Efficacy, immunogenicity, and safety of a pentavalent human-bovine (WC3) reassortant rotavirus vaccine at the end of shelf life. Pediatrics. 2007;119(1):11‑8. Available from: https://www.ncbi.nlm.nih.gov/pubmed/17200266

24. Ciarlet M, He S, Lai S, Petrecz M, Yuan G, Liu GF, et al. Concomitant use of the 3-dose oral pentavalent rotavirus vaccine with a 3-dose primary vaccination course of a diphtheria-tetanus-acellular pertussis-hepatitis B-inactivated polio-Haemophilus influenzae type b vaccine: immunogenicity and reactogenicity. Pediatr Infect Dis J. 2009;28(3):177‑81. Available from: https://www.ncbi.nlm.nih.gov/pubmed/19209092

25. Merck Sharp & Dohme Corp. Comparison of the immunogenicity and safety of three consistency lots of RotaTeq^{TM} in healthy infants (NCT00092456). Available from:

https://clinicaltrials.gov/ct2/show/record/NCT00092456; 2005.

26. Dennehy PH, Brady RC, Halperin SA, Ward RL, Alvey JC, Fischer Jr. FH, et al. Comparative evaluation of safety and immunogenicity of two dosages of an oral live attenuated human rotavirus vaccine. Pediatr Infect Dis J. 2005/06/04. 2005;24(6):481–8. Available from: https://www.ncbi.nlm.nih.gov/pubmed/15933555

27. Kim JSD, Bae CW, Lee KY, Park MS, Choi YY, Kim KN, et al. Immunogenicity, reactogenicity and safety of a human rotavirus vaccine (RIX4414) in Korean infants: a randomized, double-blind, placebo-controlled, phase IV study. Hum Vaccin Immunother. 2012;8(6):806–12. Available from: https://www.ncbi.nlm.nih.gov/pubmed/22699440

28. Kim DS, Lee TJ, Kang JH, Kim JH, Lee JH, Ma SH, et al. Immunogenicity and safety of a pentavalent human-bovine (WC3) reassortant rotavirus vaccine in healthy infants in Korea. Pediatr Infect Dis J. 2008/01/05. 2008;27(2):177–8. Available from: https://www.ncbi.nlm.nih.gov/pubmed/18174862

29. Dennehy PH, North American Human Rotavirus Vaccine Study G. A short report on the highlights of world-wide development of RIX4414: a North American experience comparative evaluation of safety and immunogenicity of two dosages of an oral live attenuated human rotavirus vaccine (RIX4414) in infants in the United Sta. Vaccine [Internet]. 2006;24(18):3780–1. Available from: https://www.ncbi.nlm.nih.gov/pubmed/16111790

30. Vesikari T, Karvonen A, Korhonen T, Espo M, Lebacq E, Forster J, et al. Safety and immunogenicity of RIX4414 live attenuated human rotavirus vaccine in adults, toddlers and previously uninfected infants. Vaccine [Internet]. 2004/07/13. 2004 Jul 29 [cited 2021 Sep 27];22(21–22):2836–42. Available from: https://www.ncbi.nlm.nih.gov/pubmed/15246619

31. Clark HF, Burke CJ, Volkin DB, Offit P, Ward RL, Bresee JS, et al. Safety, immunogenicity and efficacy in healthy infants of G1 and G2 human reassortant rotavirus vaccine in a new stabilizer/buffer liquid formulation. Pediatr Infect Dis J [Internet]. 2003;22(10):914–20. Available from: https://www.ncbi.nlm.nih.gov/pubmed/14551493

32. Clark HF, Bernstein DI, Dennehy PH, Offit P, Pichichero M, Treanor J, et al. Safety, efficacy, and immunogenicity of a live, quadrivalent human-bovine reassortant rotavirus vaccine in healthy infants. J Pediatr [Internet]. 2004/02/05. 2004;144(2):184–90. Available from: https://www.ncbi.nlm.nih.gov/pubmed/14760258

33. Jaime Caro J, Eddy DM, Kan H, Kaltz C, Patel B, Eldessouki R, et al. Questionnaire to assess relevance and credibility of modeling studies for informing health care decision making: An ISPOR-AMCP-NPC good practice task force report. Value Heal [Internet]. 2014;17(2):174–82. Available from: http://dx.doi.org/10.1016/j.jval.2014.01.003

34. Bilcke J, Van Damme P, Beutels P. Cost-effectiveness of rotavirus vaccination: Exploring caregiver(s) and "no medical care" disease impact in Belgium. Med Decis Mak [Internet]. 2009 Jan [cited 2021 Jan 13];29(1):33–50. Available from: https://pubmed.ncbi.nlm.nih.gov/18948433/

35. Perez-Rubio A, Luquero FJ, Eiros Bouza JM, Castrodeza Sanz JJ, Bachiller Luque MR, de Lejarazu RO, et al. Socio-economic modelling of rotavirus vaccination in Castilla y Leon, Spain. Le Infez Med [Internet]. 2011;19(3):166–75. Available from: http://ovidsp.ovid.com/ovidweb.cgi?T=JS&PAGE=reference&D=med8&NEWS=N&AN=22037437

36. Imaz I, Rubio B, Cornejo AM, Gonzalez-Enriquez J, González-Enríquez J. Budget impact and cost-utility analysis of universal infant rotavirus vaccination in Spain. Prev Med (Baltim) [Internet]. 2013/12/24. 2014;61:116–21. Available from: https://www.ncbi.nlm.nih.gov/pubmed/24360847

37. Yamin D, Atkins KE, Remy V, Galvani AP. Cost-Effectiveness of Rotavirus Vaccination in France. Accounting for Indirect Protection. Value Heal. 2016;19(6):811–9.

38. Goossens LMA, Standaert B, Hartwig N, Hövels AM, Al MJ. The cost-utility of rotavirus vaccination with Rotarix™ (RIX4414) in the Netherlands. Vaccine [Internet]. 2008 Feb 20 [cited 2021 Jan 13];26(8):1118–27. Available from: https://pubmed.ncbi.nlm.nih.gov/18215445/

39. Rozenbaum MH, Mangen MJJ, Giaquinto C, Wilschut JC, Hak E, Postma MJ. Cost-effectiveness of rotavirus vaccination in the Netherlands; The results of a consensus model. BMC Public Health [Internet]. 2011 Dec 10 [cited 2021 Jan 13];11(1):462. Available from: http://bmcpublichealth.biomedcentral.com/articles/10.1186/1471-2458-11-462

40. Bruijning-Verhagen P, van Dongen JAP, Verberk JDM, Pijnacker R, van Gaalen RD, Klinkenberg D, et al. Updated cost-effectiveness and risk-benefit analysis of two infant rotavirus vaccination strategies in a high-income, low-endemic setting. BMC Med [Internet]. 2018 [cited 2020 Apr 20];16(1):1–15. Available from: https://pubmed.ncbi.nlm.nih.gov/30196794/

41. Tilson L, Jit M, Schmitz S, Walsh C, Garvey P, McKeown P, et al. Cost-effectiveness of universal rotavirus vaccination in reducing rotavirus gastroenteritis in Ireland. Vaccine [Internet]. 2011 [cited 2021 Jan 13];29(43):7463–73. Available from: https://pubmed.ncbi.nlm.nih.gov/21821085/

42. Chodick G, Waisbourd-Zinman O, Shalev V, Kokia E, Rabinovich M, Ashkenazi S. Potential impact and cost-effectiveness analysis of rotavirus vaccination of children in Israel. Eur J Public Health [Internet]. 2009 Jun [cited 2021 Jan 13];19(3):254–9. Available from: https://pubmed.ncbi.nlm.nih.gov/19221026/

43. Panatto D, Amicizia D, Ansaldi F, Marocco A, Marchetti F, Bamfi F, et al. Burden of rotavirus disease and cost-effectiveness of universal vaccination in the Province of Genoa (Northern Italy). Vaccine [Internet]. 2009 [cited 2021 Jan 13];27(25–26):3450–3. Available from: https://pubmed.ncbi.nlm.nih.gov/19200850/

44. Sato T, Nakagomi T, Nakagomi O. Cost-effectiveness analysis of a universal rotavirus immunization program in Japan. Jpn J Infect Dis. 2011;64(4):277–83.

45. Itzler R, O' Brien MA, Yamabe K, Abe M, Dhankhar P. Cost-effectiveness of a pentavalent rotavirus vaccine in Japan. J Med Econ [Internet]. 2013;16(10):1216–27. Available from: https://pubmed.ncbi.nlm.nih.gov/23919721/

46. Hansen Edwards C, De Blasio BF, Salamanca BV, Flem E. Re–evaluation of the cost–effectiveness and effects of childhood rotavirus vaccination in Norway. PLoS One [Internet]. 2017 [cited 2021 Jan 13];12(8):1–19. Available from: https://pubmed.ncbi.nlm.nih.gov/28817621/

47. Milne RJ, Grimwood K. Budget impact and cost-effectiveness of including a pentavalent rotavirus vaccine in the New Zealand childhood immunization schedule. Value Heal. 2009;12(6):888–98.

48. Jit M, Edmunds WJ. Evaluating rotavirus vaccination in England and Wales. Part II. The potential cost-effectiveness of vaccination. Vaccine [Internet]. 2007 May 16 [cited 2021 Jan 13];25(20):3971–9. Available from: https://pubmed.ncbi.nlm.nih.gov/17400341/

49. Martin A, Batty A, Roberts JA, Standaert B. Cost-effectiveness of infant vaccination with RIX4414 (Rotarix™) in the UK. Vaccine. 2009;27(33):4520–8.

50. Burnett E, Parashar UD, Tate JE. Real-world effectiveness of rotavirus vaccines, 2006-19: a literature review and meta-analysis. Lancet Glob Heal [Internet]. 2020 Sep;8(9):e1195–202. Available from: https://pubmed.ncbi.nlm.nih.gov/32827481

51. Wang Y, Li J, Dai P, Liu P, Zhu F. Effectiveness of the oral human attenuated pentavalent rotavirus vaccine (RotaTeq™) postlicensure: a meta-analysis−2006–2020. Expert Rev Vaccines [Internet]. 2021 Apr 3 [cited 2021 May 11];20(4):437–48. Available from:

https://doi.org/10.1080/14760584.2021.1902808

ANNEX 1. Search strategy

Search strategy for safety, efficacy and effectiveness studies

Medline (Search date: 27-April-2020		
Number	Question	Results
1	Rotavirus/	8695
2	Rotavirus Infections/	7707
3	1 or 2	11390
4	rotavirus*.ti,ab,kw.	14699
5	(rotavirus* adj2 infection*).ti,ab,kw.	3161
6	(rotaviral adj2 infection*).ti,ab,kw.	134
7	(Neonatal adj4 Calf adj4 Diarrhea).ti,ab,kw.	143
8	4 or 5 or 6 or 7	14821
9	Rotavirus Vaccines/	2294
10	Vaccines, Attenuated/	11697
11	9 or 10	13318
12	(rotavirus adj2 vaccin*).ti,ab,kw.	3468
13	(attenuated adj2 vaccine*).ti,ab,kw.	6752
14	RotaTeq.ti,ab,kw.	371
15	Rotarix.ti,ab,kw.	446
16	RV5.ti,ab,kw.	342
17	RV1.ti,ab,kw.	228
18	vaccin*.ti,ab,kw.	308663
19	Immuni*.ti,ab,kw.	308498
20	(pentavalent adj4 rotavirus adj4 vaccin*).ti,ab,kw.	210
21	9 or 10 or 11 or 12 or 13 or 14 or 15 or 16 or 17	524773
22	3 or 8	15519
23	11 or 21	525601
24	22 and 23	5519
25	limit 24 to yr="2018 -Current"	808
26	limit 25 to humans	423

EMBASE (Search date: 27-April-2020)		
Number	Question	Results
1	'rotavirus'/exp	14019
2	'rotavirus infection'/exp	3874
3	#1 OR #2	16298
4	rotavirus*:ti,ab,kw	17164
5	(rotavirus* NEAR/2 infection*):ti,ab,kw	3748

6	(rotaviral NEAR/2 infection*):ti,ab,kw	159
7	(neonatal NEAR/4 calf NEAR/4 diarrhea):ti,ab,kw	155
8	#4 OR #5 OR #6 OR #7	17291
9	#3 OR #8	19753
10	'rotavirus vaccine'/exp	5446
11	(rotavirus NEAR/2 vaccin*):ti,ab,kw	4140
12	(attenuated NEAR/2 vaccine*):ti,ab,kw	8392
13	RotaTeq:ti,ab,kw	453
14	rotarix:ti,ab,kw	557
15	rv1:ti,ab,kw	303
16	rv5:ti,ab,kw	361
17	vaccin*:ti,ab,kw	392863
18	immuni*:ti,ab,kw	415898
19	(pentavalent NEAR/4 rotavirus NEAR/4 vaccin*):ti,ab,kw	261
20	#10 OR #11 OR #12 OR #13 OR #14 OR #15 OR #16 OR #17 OR #18 OR #19	690736
21	#9 AND #20	7332
22	#9 AND #20 AND [2018-2020]/py	1052
23	#9 AND #20 AND [2018-2020]/py AND [humans]/lim	900

Cochrane Library (Search date: 27-April-2020)		
Number	Question	Results
1	MeSH descriptor: [Rotavirus] explode all trees	229
2	MeSH descriptor: [Rotavirus Infections] explode all trees	101
3	#1 OR #2	292
4	(rotavirus*):ti	953
5	(rotavirus* NEAR/2 infection*):ti,ab,kw	467
6	(rotaviral NEAR/2 infection*):ti,ab,kw	4
7	(neonatal NEAR/4 calf NEAR/4 diarrhea):ti,ab,kw	1
8	#4 OR #5 OR #6 OR #7	954
9	#3 OR #8	954
10	MeSH descriptor: [Rotavirus Vaccines] explode all trees	217
11	MeSH descriptor: [Vaccines Attenuated] explode all trees	722
12	#10 OR #11	789
13	(rotavirus NEAR/2 vaccin*):ti,ab,kw	553
14	(attenuated NEAR/2 vaccine*):ti,ab,kw	1122
15	RotaTeq:ti,ab,kw	59
16	rotarix:ti,ab,kw	119
17	rv1:ti,ab,kw	26
18	rv5:ti,ab,kw	49
19	vaccin*:ti,ab,kw	24432
20	immuni*:ti,ab,kw	14862

21	(pentavalent NEAR/4 rotavirus NEAR/4 vaccin*):ti,ab,kw	86
22	#13 OR #14 OR #15 OR #16 OR #17 OR #18 OR #19 OR #20 OR #21	30708
23	#12 AND #22 with Cochrane Library publication date Between Apr 2018 and Apr 2020	55

Search strategy for economic studies

Medline (Search strategies: 27-April-2020		
Number	Question	Results
1	Rotavirus/	8695
2	Rotavirus Infections/	7707
3	1 or 2	11390
4	rotavirus*.ti,ab,kw.	14699
5	(rotavirus* adj2 infection*).ti,ab,kw.	3161
6	(rotaviral adj2 infection*).ti,ab,kw.	134
7	(Neonatal adj4 Calf adj4 Diarrhea).ti,ab,kw.	143
8	4 or 5 or 6 or 7	14821
9	Rotavirus Vaccines/	2294
10	Vaccines, Attenuated/	11697
11	9 or 10	13318
12	(rotavirus adj2 vaccin*).ti,ab,kw.	3468
13	(attenuated adj2 vaccine*).ti,ab,kw.	6752
14	RotaTeq.ti,ab,kw.	371
15	Rotarix.ti,ab,kw.	446
16	RV5.ti,ab,kw.	342
17	RV1.ti,ab,kw.	228
18	vaccin*.ti,ab,kw.	308663
19	Immuni*.ti,ab,kw.	308498
20	(pentavalent adj4 rotavirus adj4 vaccin*).ti,ab,kw.	210
21	9 or 10 or 11 or 12 or 13 or 14 or 15 or 16 or 17	524773
22	3 or 8	15519
23	11 or 21	525601
24	22 and 23	5519
25	limit 25 to humans	4235
26	Economics, Nursing/ or Economics, Pharmaceutical/ or Economics/ or Economics, Medical/ or Economics, Hospital/	53627
28	Cost-Benefit Analysis/	80206
29	Drug Costs/ or Health Care Costs/ or Models, Economic/	60697
30	Budgets/	11265
31	economic*.ti,ab,kw.	278872
32	pharmacoeconomic*.ti,ab,kw.	3902
33	cost*.ti,ab,kw.	599044

34	pric*.ti,ab,kw.	55341
35	cost-effectiveness.ti,ab,kw.	61424
36	cost-utility.ti,ab,kw.	4625
37	cost-benefit.ti,ab,kw.	10078
38	Incremental adj4 Cost adj4 Effectiveness adj4 Ratio.ti,ab,kw.	5269
39	INB.ti,ab,kw.	181
40	Disability adj4 Adjusted adj4 Life adj4 Years.ti,ab,kw.	2708
41	DALY.ti,ab,kw.	1763
42	Quality adj4 Adjusted adj4 Life adj4 Years.ti,ab,kw.	7913
43	QALY.ti,ab,kw.	8602
44	26 or 27 or 28 or 29 or 30 or 31 or 32 or 33 or 34 or 35 or 36 or 37 or 38 or 39 or 40 or 41 or 42 or43	917673
45	25 and 44	642
46	limit 45 to yr="2015 -Current"	181

EMBASE (Search date: 27-April-2020)		
Number	Question	Results
1	'rotavirus'/exp	14019
2	'rotavirus infection'/exp	3874
3	#1 OR #2	16298
4	rotavirus*:ti,ab,kw	17164
5	(rotavirus* NEAR/2 infection*):ti,ab,kw	3748
6	(rotaviral NEAR/2 infection*):ti,ab,kw	159
7	(neonatal NEAR/4 calf NEAR/4 diarrhea):ti,ab,kw	155
8	#4 OR #5 OR #6 OR #7	17291
9	#3 OR #8	19753
10	'rotavirus vaccine'/exp	5446
11	(rotavirus NEAR/2 vaccin*):ti,ab,kw	4140
12	(attenuated NEAR/2 vaccine*):ti,ab,kw	8392
13	RotaTeq:ti,ab,kw	453
14	rotarix:ti,ab,kw	557
15	rv1:ti,ab,kw	303
16	rv5:ti,ab,kw	361
17	vaccin*:ti,ab,kw	392863
18	immuni*:ti,ab,kw	415898
19	(pentavalent NEAR/4 rotavirus NEAR/4 vaccin*):ti,ab,kw	261
20	#10 OR #11 OR #12 OR #13 OR #14 OR #15 OR #16 OR #17 OR #18 OR #19	690736
21	#9 AND #20	7332
22	#9 AND #20 AND [humans]/lim	5848
23	'socioeconomics'/exp OR 'socioeconomics'	385200
24	'cost benefit analysis'/exp	83593
25	'cost effectiveness analysis'/exp	149413

26	'cost of illness'/exp	18879
27	'cost control'/exp	67637
28	'financial management'/exp	435938
29	'health economics'/exp	855708
30	'health care cost'/exp	289526
31	'health care financing'/exp	13235
32	economic*:ti,ab,kw	352217
33	pharmacoeconomic*:ti,ab,kw	8833
34	cost*:ti,ab,kw	821570
35	pric*:ti,ab,kw	86857
36	'cost effectiveness':ti,ab,kw	86560
37	'cost utility':ti,ab,kw	7590
38	'cost benefit':ti,ab,kw	15648
39	(incremental NEAR/4 cost NEAR/4 effectiveness NEAR/4 ratio):ti,ab,kw	8888
40	icer:ti,ab,kw	9029
41	(incremental NEAR/3 net NEAR/3 benefit):ti,ab,kw	339
42	int:ti,ab,kw	22927
43	(disability NEAR/4 adjusted NEAR/4 life NEAR/4 years):ti,ab,kw	3363
44	(quality NEAR/4 adjusted NEAR/4 life NEAR/4 years):ti,ab,kw	12763
45	qaly:ti,ab,kw	16319
46	daly:ti,ab,kw	2357
47	#26 OR #27 OR #28 OR #29 OR #30 OR #31 OR #32 OR #33 OR #34 OR #35 OR #36 OR #37 OR #38 OR #39 OR #40 OR #41 OR #42 OR #43 OR #44 OR #45 OR #46 OR #47 OR #48	1907640
48	#22 AND #47	1336
49	#22 AND #47 AND [2015-2020]/py	408

Paediatric Economic Database Evaluation (Search date: 27-April-2020)		
Number	Question	Results
1	(TITLE_ABSTRACT_KEYWORDS "rotavirus")<br>Age groups: Perinates Neonates Infants Children Adolescents<br>Years: 2015 - 2018	31

ANNEX 2. Systematic reviews AMSTAR 2 quality assessment

	Soares 2019 (2)	Kotirum 2017 (3)	Burnett 2020 (50)	Wang 2021 (51)	Sun 2021 (6)	Rha 2014 (9)	Gidengil 2021 (7)	Kassim 2017 (8)	Mellone 2019 (10)
**1. Did the research questions and inclusion criteria for the review include the components of PICO?**	Yes	Yes	Yes	Yes	Yes	No	Yes	Yes	Yes
**2. Did the report of the review contain an explicit statement that the review methods were established prior to the conduct of the review and did the report justify any significant deviations from the protocol?**	Yes	Partial Yes	No	No	No	No	Partial Yes	Partial Yes	Partial Yes
**3. Did the review authors explain their selection of the study designs for inclusion in the review?**	Yes	Yes	Yes	Yes	Yes	No	Yes	Yes	No
**4. Did the review authors use a comprehensive literature search strategy?**	Partial Yes	Partial Yes	No	Partial Yes	Partial Yes	No	Yes	Partial Yes	Yes
**5. Did the review authors perform study selection in duplicate?**	Yes	Yes	No	Yes	Yes		Yes	Yes	Yes
**6. Did the review authors perform data extraction in duplicate?**	Yes	Yes	No	No	Yes	No	No	No	Yes
**7. Did the review authors provide a list of excluded studies and justify the exclusions?**	Yes	No	No	No	No	No	Partial Yes	No	Partial Yes
**8. Did the review authors describe the included studies in adequate detail?**	Yes	Yes	Partial Yes	Yes	Yes	Partial Yes	Yes	Yes	Yes
**9. Did the review authors use a satisfactory technique for assessing the risk of bias (RoB) in individual studies that were included in the review?**									
**RCTs**	Yes	No	Includes only NRSI	Includes only NRSI	Yes	No	No	Includes only NRSI	Yes
**NRSI**	Includes only RCTs	No	No	Partial Yes	Partial Yes	No	No	Partial Yes	Partial Yes
**10. Did the review authors report on the sources of funding for the studies included in the review?**	Yes	Yes	No	No	No	No	Yes	No	No
**11. If meta-analysis was performed did the review authors use appropriate methods for statistical combination of results?**									
**RCTs**	Yes	No meta-analysis	Includes only NRSI	Includes only NRSI	No				Yes
**NRSI**	No meta-analysis conducted	No meta-analysis conducted	Yes	Yes	No		No	No	No

	Soares 2019 (2)	Kotirum 2017 (3)	Burnett 2020 (50)	Wang 2021 (51)	Sun 2021 (6)	Rha 2014 (9)	Gidengil 2021 (7)	Kassim 2017 (8)	Mellone 2019 (10)
**12. If meta-analysis was performed, did the review authors assess the potential impact of RoB in individual studies on the results of the meta-analysis or other evidence synthesis?**	No	No meta-analysis	No	Yes	Yes		No	No	No
**13. Did the review authors account for RoB in individual studies when interpreting/ discussing the results of the review?**	Yes	No	No	Yes	No	Yes	Yes	Yes	Yes
**14. Did the review authors provide a satisfactory explanation for, and discussion of, any heterogeneity observed in the results of the review?**	Yes	No	Yes	Yes	Yes		No	No	Yes
**15. If they performed quantitative synthesis did the review authors carry out an adequate investigation of publication bias (small study bias) and discuss its likely impact on the results of the review?**	No	No meta-analysis conducted	Yes	Yes	Yes		Yes	Yes	No
**16. Did the review authors report any potential sources of conflict of interest, including any funding they received for conducting the review?**	Yes	Yes	Yes	Yes	Yes	Yes	Yes	No	Yes
